# Improved performance of small molecule organic solar cells by incorporation of a glancing angle deposited donor layer

**DOI:** 10.1038/s41598-020-62769-3

**Published:** 2020-04-01

**Authors:** Qi Jiang, Yingjie Xing

**Affiliations:** Key Laboratory for the Physics and Chemistry of Nanodevices, Beijing Key Laboratory of Quantum Devices, and Department of Electronics, Peking University, Beijing, 100871 China

**Keywords:** Energy science and technology, Optics and photonics

## Abstract

Improving the photovoltaic performance directly by innovative device architectures contributes much progress in the field of organic solar cells. Photovoltaic device using different kinds of heterojunction with the given set of organic semiconductors paves the way to a better understanding of the working mechanism of organic heterojunction. Here, we report on the fabrication of a new device structure without employing extra material. A thin film of the donor material (chloroaluminum phthalocyanine (ClAlPc)) is inserted between ClAlPc:C60 bulk heterojunction (BHJ) and C60 layer by glancing angle deposition. A ClAlPc/C60 planar heterojunction co-exists with ClAlPc:C60 BHJ simultaneously in this device. Higher efficiency is obtained with this novel device structure. The effects of this additional ClAlPc layer on open-circuit voltage and fill factor in photovoltaic cells are studied. This work provides a new route to improve the device performance of organic solar cells.

## Introduction

As the first prototype of organic solar cell, small molecule organic photovoltaic device has attracted intensive research interest since three decades ago^1^. Photovoltaic cells with metal phthalocyanine as the donor and fullerene as the acceptor have shown good performance and are long been regarded as an ideal candidate for studying the working mechanism of organic solar cell^2,3^. As more and more novel donor/acceptor small molecules are designed and synthesized, a continuous enhancement of energy conversion efficiency is achieved due to broader optical absorption and more efficient charge transport in small molecule photovoltaic devices^3–6^.

Comparing to the significant progresses in molecular synthesis, less improvements are obtained in device architecture of small molecule organic solar cell^1,7,8^. Planar heterojunction (PHJ) is the first kind of device structure of organic photovoltaic device^9^, but this structure is rarely employed nowadays because the pristine PHJ device usually shows poor efficiency^1^. The efforts to modify the device structure beyond PHJ create two kinds of novel donor/acceptor interface. While still keeping two unmixed donor and acceptor regions, a rough interface between donor and acceptor formed by glancing angle deposition (GLAD) technique, improves the conversion efficiency significantly^10,11^. Both efficient charge transport in pure donor/acceptor region and larger interface area for exciton dissociation are proposed as the benefits due to this rough and unmixed interface. The other innovative structure is the molecular scale mixture of donor and acceptor, which is called bulk heterojunction (BHJ). BHJ is the most popular device structure in the field of organic photovoltaic device, and almost all high efficiency organic solar cells adopt this structure. In small molecule solar cells, BHJ is usually sandwiched between a donor layer and an acceptor layer, which is called p-i-n structure. It is found that three factors of devices in p-i-n structure, i.e., short-circuit density (J_sc_), open-circuit voltage (V_oc_), and fill factor (FF), are mainly determined by BHJ. High efficiency is obtained when the balance of more optical absorption and less charge loss is approached by optimizing the film thickness and the D/A ratio of BHJ accurately^1^.

The aim of most above discussed efforts, including both molecule synthesis and BHJ optimization, focuses on how to obtain higher J_sc_, V_oc_, and FF by tuning the “internal” property of BHJ. For example, because V_oc_ is generally believed to be limited by a voltage difference between the highest occupied molecular orbital (HOMO) level of the donor and the lowest unoccupied molecular orbital (LUMO) level of the acceptor, a larger V_oc_ is hoped by employing novel donor-acceptor molecules with larger voltage difference^12^. In addition to various studies to adjust the “internal” property of BHJ devices directly, there are some other attempts to indirectly improve the device performance by imposing “external” influence on BHJ, e.g., more optical absorption due to proper design of light trapping configurations^13,14^.

The success of BHJ has been proved by the significant enhancement of J_sc_ comparing with that in PHJ devices. However, FF and V_oc_ in BHJ devices, the other two factors determining the efficiency, are difficult to improve continuously due to the restriction of the “internal” property of BHJ. It is well known that FF is easily influenced by some intrinsic factors in BHJ, e.g., randomly mixed morphology, unbalanced mobility of donor and acceptor, and bimolecular recombination^15^. For V_oc_, a tradeoff between the achievable V_oc_ and the charge carrier generation efficiency is found in many BHJ devices. That is to say, if a larger V_oc_ is obtained by using novel donor and acceptor molecules with more optimal energy level offset, the driving force for charge generation in BHJ will be weakened inevitably^16,17^. This contradiction reveals another intrinsic limitation coming from the “internal” property of BHJ. Here, we improve V_oc_ and FF of small molecule BHJ photovoltaic devices by an “external” factor. We change the interface between BHJ (i) and the acceptor layer (n) in common p-i-n structure and produce a novel p-i-p-n structure. A thin donor layer is intentionally deposited behind the BHJ layer as the second p layer, resulting in an extra PHJ (p-n). Chloroaluminum phthalocyanine (ClAlPc), a kind of metal phthalocyanine with optical absorption in near-infrared band^18^, is used as the donor in this work. We obtain higher efficiency in ClAlPc/ClAlPc:C60/ClAlPc/C60/bathocuproine (BCP)/Al device. The effect of the second ClAlPc layer on the photovoltaic performance is investigated detailedly. It is found that higher efficiency of p-i-p-n structure comes from ClAlPc/C60 PHJ with a proper build-in field. To the best of our knowledge, this result is the first report on the effect of an extra build-in field on BHJ inside a photovoltaic cell. Our work shows the property of D/A interface in equilibrium state can be used to improve the performance of BHJ in non-equilibrium in organic photovoltaic devices.

## Methods

### Device fabrication and measurement

Cleaned indium tin oxide (ITO) glasses are used as the substrates in all devices. ClAlPc (Yannuo Chem., purity 99%) is used as the donor material. The chemical structure of ClAlPc is shown in the Supplementary Information. Fullerene C60 (Yannuo Chem., purity 99%) is used as the acceptor material. BCP (Yannuo Chem., purity 99%) is used as the cathode buffer material. All above materials are used as received. The donor:accepter (D/A) mixtures are all in weight ratio 1:1. The devices are fabricated in a commercial vacuum deposition system (ULVAC-KIKO VWR-400M/ERH). The procedure of device fabrication is similar to our previous experiment^19^. Organic materials are deposited onto ITO substrates successively at a rate of ~0.05 nm/s under a pressure of ~1 × 10^−4^ Pa. Aluminum is deposited at a rate of ~0.5 nm/s under a pressure of 5 × 10^−3^ Pa. The film thickness and deposition rate are monitored *in situ* using a quartz crystal oscillator. The area of a single device is 4 mm^2^. At least three batches of photovoltaic devices are prepared under each condition. The best efficiency in each batch is used to compare in this work. A sunlight simulator (Newport Oriel 91160) is used to illuminate the sample with the power of 100 mW/cm^2^. The current-voltage (I-V) curves are measured by an electrochemical analyzer. All I-V measurements are performed in air. Optical absorption is measured with a UV-Vis-NIR spectrophotometer (Cary 5000) in air. Atomic force microscope (AFM, DI NanoScope) is used to scan the surface of GLAD deposited ClAlPc film.

**Figure 1:**
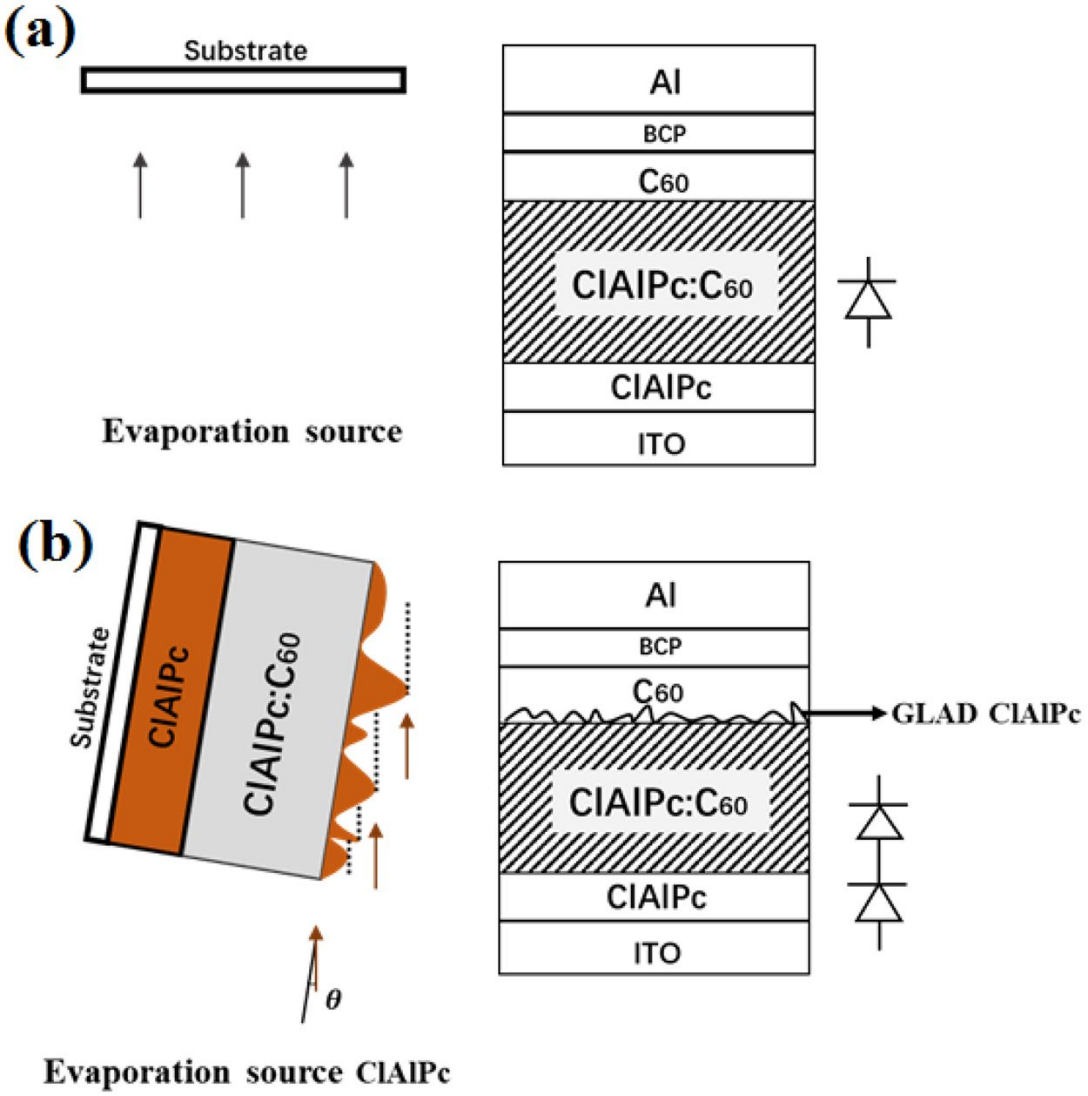
Schematic illustration of two kinds of deposition style. (**a**) normal deposition (type I devices, ClAlPc/ClAlPc:C60 and C60/BCP/Al layers in type II devices), (**b**) glancing angle deposition (the second ClAlPc layer in type II devices). Device structure and schematically device model of type I and II devices are also shown.

Two types of device architecture are prepared in our experiments. The first type (p-i-n), ClAlPc/ClAlPc:C60/C60/BCP/Al, is prepared by normal deposition, in which the evaporation flux reaches the substrate perpendicularly. Type I devices have the thicknesses of ClAlPc (10 nm)/ClAlPc:C60 (40 nm)/C60 (10 nm)/BCP (8 nm)/Al (80 nm). For the second type (p-i-p-n), ClAlPc/ClAlPc:C60/ClAlPc/C60/BCP/Al, GLAD is used to deposit an additional ClAlPc layer behind ClAlPc:C60 BHJ, and normal deposition is used to deposit all other layers. The angle of the substrate relative to the incident beam is tuned in the vacuum chamber *in situ* by manual operation^20^. Tuning degree is approximately 85°. Fig. 1a,b show two kinds of deposition schematically. The actual thickness of GLAD deposited ClAlPc layer cannot be determined by the quartz crystal oscillator directly. We try to determine the thickness by AFM but fail because the GLAD film is rather coarse and thin. Hereafter, we use the monitored thickness as a relative indicator to compare the thickness of ClAlPc layer deposited in glancing angle. We carefully optimize the thicknesses of ClAlPc (GLAD) film and two layers next to it (ClAlPc:C60 and C60). The thickness of other layer in type II device is maintained as the same value as in type I device. The optimal type II devices have the thicknesses of ClAlPc (10 nm)/ClAlPc:C60 (30 nm)/ClAlPc (GLAD, 20 nm)/C60 (15 nm)/BCP (8 nm)/Al (80 nm). The calculated thickness of GLAD deposited ClAlPc layer with a monitored thickness of 20 nm is estimated to be approximately 2 nm according to the trigonometric relation. We note that the scanned morphology by AFM measurement deviates from this value (2 nm) significantly. Detailed result of characterization of GLAD deposited ClAlPc will be shown in following section.

### Field emission measurement

A home-made field emission microscope is designed and fabricated to investigate the band bending in organic PHJ. The base pressure of field emission microscope is ~2 × 10^−7^ Pa. ClAlPc/C60 PHJ is deposited layer-by-layer on a tungsten tip and field emission measurement is conducted *in situ* in sequence to measure the thickness dependence of work function. Each layer has a thickness of 1–2 nm. The total thickness of the organic PHJ on top of the bare W tip is ~15 nm. The field emission from the fresh deposited surface is quite stable without the influence of absorption. The voltage applied on the tip is limited to make sure a small emission current (<5 × 10^−6^ A). The details of the field emission measurement can be found in ref. ^21^. and the Supplementary Information.

## Results and discussion

The potential of ClAlPc as a candidate of near-infrared sensitizer has been studied and a growth rate dependent efficiency is observed in ClAlPc/C60 PHJ devices^18^. Further improvement of the performance of ClAlPc/C60 PHJ and ClAlPc:C60 BHJ devices is achieved by using anode buffer layer and heated substrate holder to initiate a template growth of ClAlPc film^22^. The oriented ClAlPc film formed under dedicated conditions is found to be the key factor for the better photovoltaic performance than that in the pristine devices with amorphous ClAlPc layer grown without bottom template molecules and at room temperature. In addition to above method of introducing an oriented ClAlPc film, it is still desirable to find other approach independent to the template material and substrate temperature to improve the efficiency of ClAlPc:C60 BHJ device, which can provide more insight to the “internal” property of BHJ.

**Figure 2:**
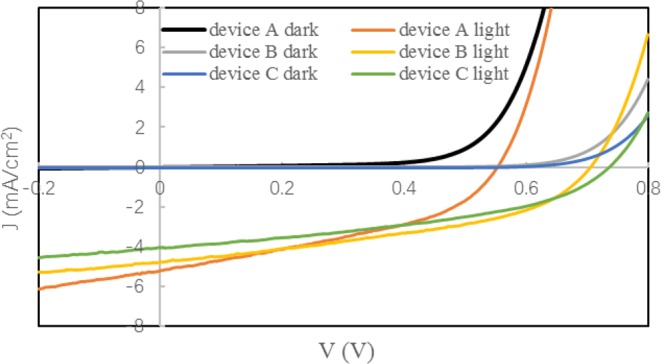
Current density-voltage (J-V) curves of standard device A modified device B and device C in the dark and under light illumination.

We fabricate ClAlPc/ClAlPc:C60/C60/BCP/Al device firstly. ClAlPc:C60 BHJ layers with different thickness and D/A ratio are deposited and the corresponding efficiencies are compared. The optimized ClAlPc:C60 BHJ has a thickness of 40 nm and a D/A ratio of 1:1. The best device shows an efficiency of 1.16%. We call this device as standard device A. The parameters of this device are listed in Table 1 and the measured current density-voltage (J-V) curve is plotted in Fig. 2. We try to further improve the device performance beyond the optimization technique.

**Table 1:** Photovoltaic performance and extracted parameters of devices.

Device No.	J_sc_ (mA/cm^2^)	V_oc_ (V)	FF	PCE	R_s_ (Ω/cm^2^)	R_sh_ (Ω/cm^2^)	n
Standard device A	5.20	0.55	0.40	1.16%	0.98	222.74	1.85
Modified device B	4.80	0.71	0.43	1.46%	0.46	395.19	2.59
Type II devices (average)	4.88	0.72	0.40	1.42%	0.54	347.62	2.74
Device C	4.05	0.74	0.42	1.25%	0.74	512.80	2.78

**Figure 3:**
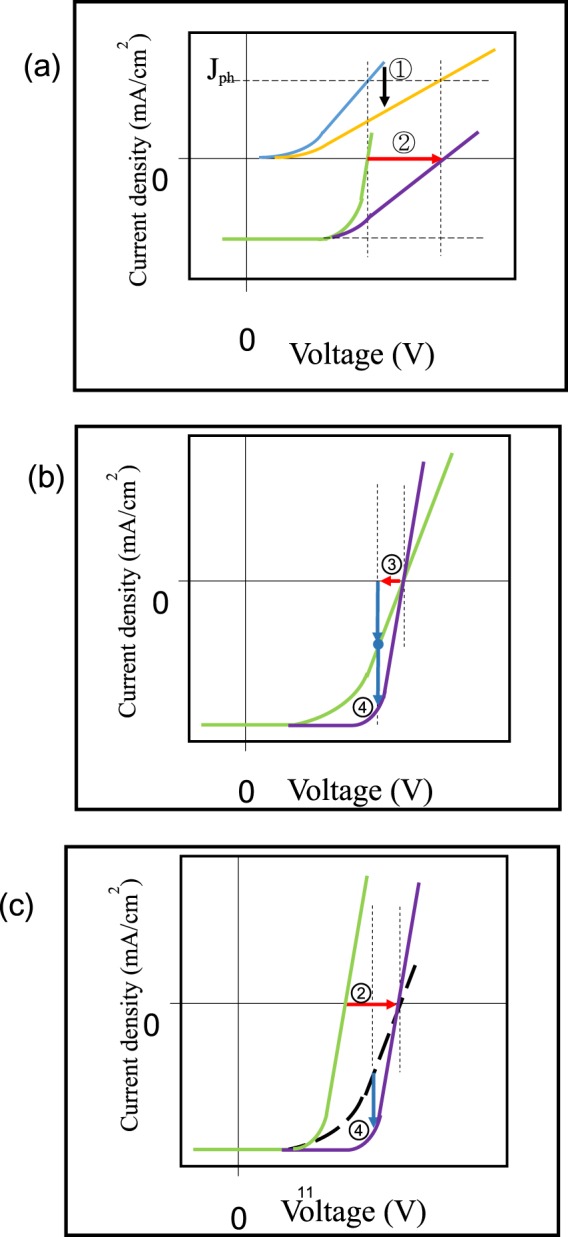
Schematic demonstration of the shape change of J-V curve considering the effects of ClAlPc/C60 PHJ. (**a**) Considering only the effect of suppressing the dark current (label ①). V_oc_ enhances correspondingly in this case (label ②). The slope of J-V curve (purple) in front of the V_oc_ point is less than that of J-V curve (green). J_ph_ is the photo-generated current density. (**b**) Considering only the effect of extracting the dissociated charges (label ③). The output current enlarges under such a small “external” field (label ④). We think that in a voltage range closing to V_oc_, the effect of a small “external” field on charge extraction is similar to a small bias deviation to the left direction from V_oc_ (marked as a “red arrow”). Supposing under a particular bias, the original output current (blue dot in green curve) is equal to the extracted current by the “external” build-in field (the upper blue arrow), then the final output current enlarges to two times of the original current, because the original current will superimpose on the extracted current (the upper blue arrow moves down). Therefore, the slope of J-V curve (purple) in front of the V_oc_ point is larger than that of J-V curve (green). (**c**) Effects of both improved V_oc_ (label ②) and enlarged output current (label ④). The slope of J-V curve (purple) in front of the V_oc_ point is similar to that of J-V curve (green). Green curve: original type I device. Black dash curve: imaginary J-V curve with only V_oc_ shift. Purple curve: modified type II device.

We have enlarged V_oc_ in zinc phthalocyanine (ZnPc): 3,4,9,10-perylene tetracarboxylic bisbenzimidazole (PTCBI) BHJ device by an extra build-in field formed in a small molecule cathode buffer^19^. Here we increase V_oc_ in ClAlPc:C60 BHJ device by a similar way. It is known that the position of V_oc_ is defined at the zero-current point, where the photocurrent equals to the injection current. This definition means that if the photocurrent does not change, V_oc_ will be enhanced by a suppressed injection current^12,23^ This method of enhancing V_oc_ is shown in Fig. 3a schematically. Although the idea of this method is simple, how to suppress the injection current but not influence the device performance under light irradiation is not facile. Based on the theory of organic-organic heterojunction^24–26^, we choose a way to suppress the injection current by introducing an additional diode-like PHJ in the photovoltaic device^19^. However, some requirements, such as energy levels between neighbor organic materials, the direction of build-in field in PHJ, and the influence of PHJ on optical absorption in original BHJ, must be considered prior to device fabrication. We use the same D/A material, i.e., ClAlPc/C60, to fabricate both PHJ and BHJ, because an additional thin ClAlPc/C60 PHJ can fulfill all above requirements. For example, a large variation of optical absorption in BHJ will not be induced by such a PHJ. The direction of build-in field and the energy levels will be discussed in the following section.

**Figure 4:**
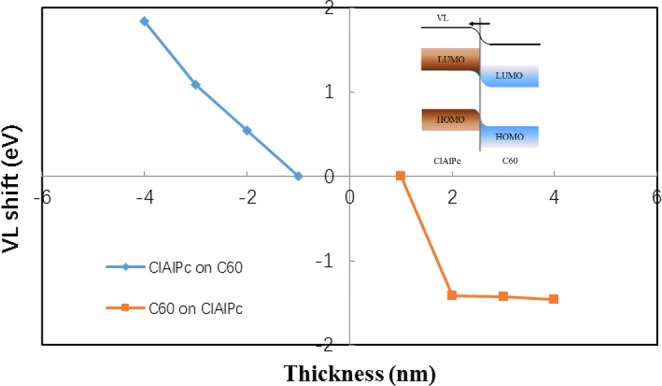
Vacuum level (VL) shift in ClAlPc/C60 heterojunction measured by field emission. Inset: Schematic demonstration of band bending and build-in field direction in ClAlPc/C60 heterojunction.

No data of the band bending in ClAlPc/C60 PHJ is reported in literature. We measure the band bending in ClAlPc/C60 PHJ by field emission. The standard Fowler-Nordheim model for field emission is employed to obtain the dependence of work function on film thickness^21^. The measured shift of vacuum level in ClAlPc/C60 PHJ is shown in Fig. 4. This shape of band bending is similar to a traditional inorganic p-n junction, indicating a build-in field with the direction from C60 (n) to ClAlPc (p). More details of the field emission measurement and calculated slopes of Fowler-Nordheim plot can be found in the Supplementary Information. We note that the build-in field in ClAlPc/C60 PHJ is generated in the equilibrium state, which means the effect of this field exists regardless of the illumination condition and the bias.

**Figure 5:**
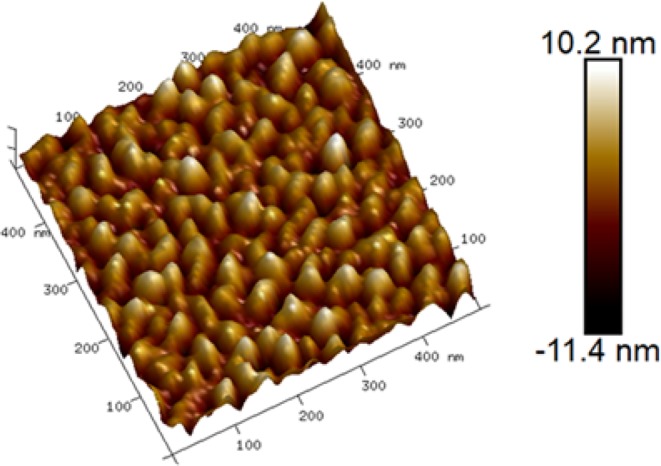
AFM image of surface morphology of ClAlPc thin film deposited in glancing angle on ITO glass. Thin and thick areas randomly distribute in the film.

Based on the direction of build-in field in ClAlPc/C60 PHJ, we propose that type II devices should have a structure of ClAlPc/ClAlPc:C60/ClAlPc/C60/BCP/Al. ClAlPc/C60 PHJ acts as an additional diode in this modified device to suppress the injection current. However, the second ClAlPc layer sandwiched between ClAlPc:C60 BHJ and C60 layer can behave as an obstacle to extracting electrons dissociated in ClAlPc:C60 BHJ. We use GLAD technique to solve this problem. It is known that the film prepared by GLAD usually has a rough surface. This phenomenon means that in a thin film deposited by GLAD, some regions may be rather thin, or even “transparent”. We use AFM to scan the surface of ClAlPc layer deposited on ITO glass by GLAD. The thickness of this film recorded by quartz crystal oscillator is ~20 nm. The AFM image is shown in Fig. 5, demonstrating a rather coarse morphology in GLAD deposited film. Calculated roughness of this film is 3.02 nm, which is larger than that of a film deposited horizontally (2.17 nm). Thin regions in GLAD deposited film provide transport routes with much less barrier for electrons.

It has been summarized that restricting the reverse dark saturation current in BHJ can increase V_oc_ in organic solar cells^12^. We note that our work is completely different from this method. The mechanism of the suppressed injection current here is due to the insertion of a diode-like PHJ, whereas for BHJ, no particular treatment is conducted to influence its reverse dark saturation current. The dark currents of type I and II device are shown in Fig. 2. The shift of dark current to the right in type II device clearly confirms the injection current is effectively suppressed by incorporation of a thin ClAlPc film. More experiment to testify the effect of ClAlPc/C60 PHJ on dark current can be found in Supplementary Information. We schematically draw device model of type I (one diode) and II devices (two diodes connected in series) in Fig. 1 according to above results. Figure 2 shows an obvious improvement of V_oc_ in a type II device under illumination as expected. The highest efficiency of type II devices is 1.46%. This device is denoted as the modified device B. More experiments are conducted to confirm the improvement of V_oc_ and efficiency. The average values of parameters of type II devices fabricated in three batches are also listed in Table 1. Comparing to standard device A, lower J_sc_, higher V_oc_, and similar FF appear after incorporation of a GLAD deposited ClAlPc layer.

**Figure 6:**
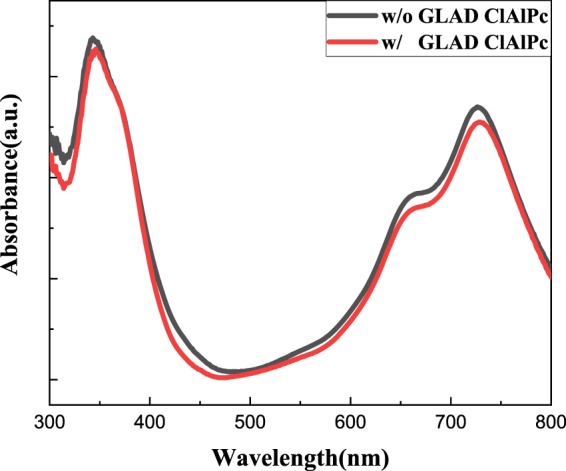
Optical absorption spectrum of ITO/ClAlPc/ClAlPc:C60/C60 and ITO/ClAlPc/ClAlPc:C60/ClAlPc(GLAD)/C60 sample.

A small reduction of J_sc_ is found in type II devices. We measure the optical absorption of ITO/ClAlPc/ClAlPc:C60/C60 and ITO/ClAlPc/ClAlPc:C60/ClAlPc/C60 sample. The absorption spectrum is shown in Fig. 6. Only slight difference is observed between two samples. Different from the single BHJ in type I device, exciton dissociation in type II device occurs in two heterojunctions: a thinner ClAlPc:C60 BHJ and an additional ClAlPc/C60 PHJ. It is known that PHJ shows much less power to dissociate excitons than BHJ. Therefore, smaller J_sc_ in type II devices reflects the decreased quantity of exciton in thinner BHJ and reduced power of exciton dissociation in PHJ.

It should be noted that the efficiency of organic solar cell can hardly be improved by above approach of suppressing the injection current itself, because just shifting V_oc_ position to the right side will deteriorate FF inevitably at the same time. Figure 3a schematically shows the interplay between V_oc_ and FF when injection current is suppressed. It can be seen that the slope of J-V curve in front of the V_oc_ point decreases obviously after the shift of V_oc_. In the present work, FF keeps almost the same value while V_oc_ enlarges, which is observed in many samples. This phenomenon reveals the charge extraction is greatly enhanced in the bias range surrounding 0.55 V (V_oc_ of standard device A) in type II devices than that in type I devices. We think that there should be the other role of ClAlPc/C60 PHJ besides suppressing the dark current, and this role compensates the deterioration effect on FF due to V_oc_ shift.

**Figure 7:**
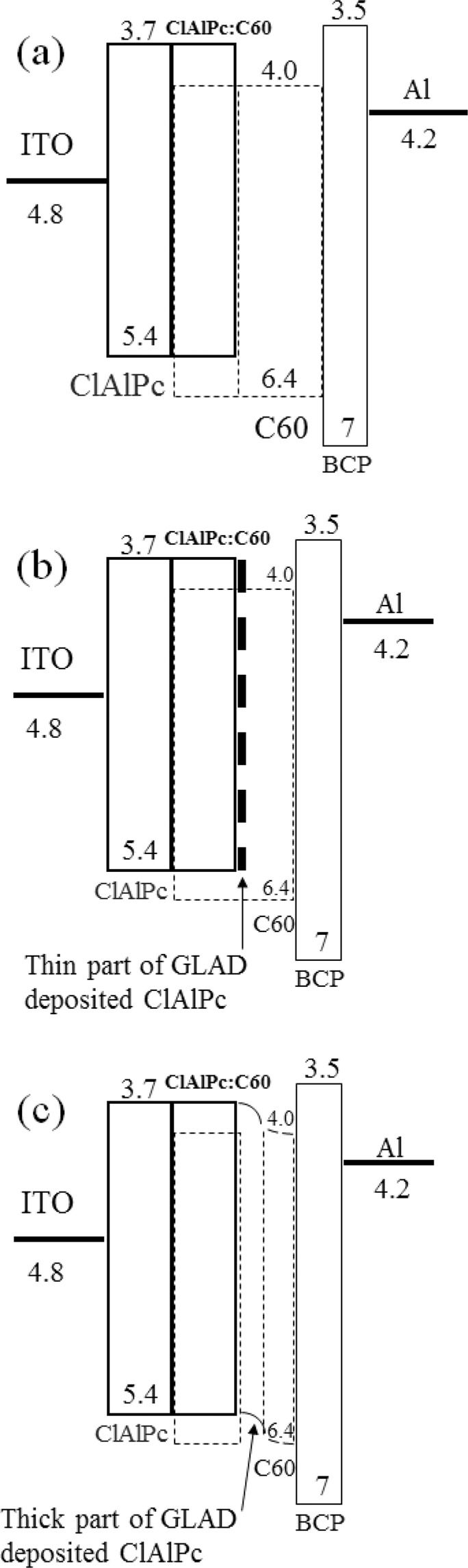
Schematic demonstration of energy level diagram. (**a**) Type I devices. (**b**) Thin part of GLAD ClAlPc layer in type II devices. The barrier effect of thin part of GLAD ClAlPc layer is negligible for electrons. (**c**) Thick part of GLAD ClAlPc layer in type II devices. Band bending occurs in this part.

Comparing to J_sc_ and V_oc_, FF is proposed to be the least understood parameter in organic solar cell^15^. There still lacks a sophisticated theory about how to calculate FF from the viewpoint of device working mechanism up to date^15^. We propose that the “external” field, generated in ClAlPc/C60 PHJ, greatly improves the charge extraction in the bias range surrounding 0.55 V in type II devices, resulting in an almost undeteriorated FF. We have shown that the build-in field in ClAlPc/C60 PHJ has the same direction with the electron extraction, and this field exists regardless of the bias and light illumination. GLAD deposited ClAlPc film is rather coarse, resulting in roughly two parts in the film: thicker than the average thickness and thinner than the average thickness. The thinner part can be regarded as more suitable sites for electron extraction from ClAlPc:C60 BHJ, whereas for the thicker part connecting C60 layer, it contributes the build-in field to extract electrons from ClAlPc:C60 BHJ. Therefore, a ClAlPc layer deposited by GLAD plays the key role in p-i-p-n structure. The energy level diagrams of type II device are shown in Fig. 7, demonstrating two kinds of band diagram in a same ClAlPc/C60 PHJ. The values of energy level in Fig. 7 are adopted from ref. ^27^.

It is known that under the open-circuit bias, i.e., 0.55 V for the standard device A, zero output current appears because all charges recombine and lose totally. A rather sensitive dependence of the charge carrier recombination/extraction on the actual field inside the active layer is therefore concluded in the bias range surrounding V_oc_ point^12^. A benefit of a proper variation of the actual field inside the active layer has been reported in such a bias range. Increased FF in small molecule organic solar cell is found because of employing an organic/low work function metal bilayer buffer with a suitable build-in field^28^. In the present work, a small “external” field exists in ClAlPc/C60 PHJ besides ClAlPc:C60 BHJ. This “external” field has a direction from C60 layer to ClAlPc:C60 BHJ, and shows a beneficial effect on electron extraction out of ClAlPc:C60 BHJ to C60 layer when the original field in the active layer is not large enough to extract electrons. This effect becomes more significant in the case of a bias range closing to 0.55 V. More extracted electrons under the “external” field than those under the original field means a larger output current, leading to a larger slope of J-V curve in front of the V_oc_ point. This effect of the build-in field in ClAlPc/C60 PHJ on FF is schematically drawn in Fig. 3b. We think that although the build-in field in ClAlPc/C60 PHJ may be neglected under a bias far from the open-circuit voltage, its effect on charge extraction is obvious in the bias range surrounding 0.55 V. Such a mechanism to improve charge extraction shows the other role of ClAlPc/C60 PHJ. In an actual type II device, FF must be influenced by both roles of ClAlPc/C60 PHJ simultaneously, resulting in an almost undeteriorated FF. Figure 3c schematically shows the shape change of J-V curve after the shift of V_oc_ and the enlargement of output current.

According to classic semiconductor device theory, FF is influenced by diode ideality factor (n), series resistance (R_s_), and shunt resistance (R_sh_)^29^. An undeteriorated FF value in type II devices seems to suggest similar device parameters as those in standard device A. We extract three device parameters (R_s_, R_sh_, n) from measured J–V curves based on a method reported in ref. ^30^. The values of n, R_s_, and R_sh_ are listed in Table 1. It can be seen that comparing to the values in standard device A, larger n, lower R_s_, and higher R_sh_ appear in type II devices. Lower R_s_ and higher R_sh_ reveal that the behavior of two connected heterojunctions (ClAlPc:C60 BHJ and ClAlPc/C60 PHJ) are more closing to an ideal photovoltaic diode than a single BHJ device. Another difference between type II devices and standard device A is the value of n. The standard device A has an ideality factor of 1.85, whereas for the type II devices, the average value of ideality factor is close to 3. A value of n between 1 and 2 in inorganic diode means the occurrence of charge recombination within the depletion region based on classic semiconductor device physics. A similar ideality factor between 1 and 2 is often extracted in organic BHJ solar cells, but the actual physical meaning of this value of n is not quite clear up to date^15^. Generally, a value equal to or less than 2 is usually recognized as a normal range of ideality factor in organic BHJ devices, although a value of n larger than 2 has been reported in some cases^31^. In the present work, an ideality factor larger than 2 appears in many type II devices. We believe that this phenomenon closely relates to the appearance of two connecting heterojunctions, i.e., ClAlPc:C60 BHJ and ClAlPc/C60 PHJ, in type II devices. High ideality factor has been studied in AlGaN/GaN diode with multiple heterojunctions for a long time^32^. It is concluded that the ideality factor extracted from measured J-V curve can larger than 2 if some extra rectifying heterojunctions exist in a true diode device. The extracted n can be roughly regarded as the sum of ideality factor in each individual heterojunction. We have observed such a phenomenon of an ideality factor larger than 2 in organic photovoltaic devices with multiple heterojunctions^19^. A value of n larger than 2 is plausible when ClAlPc:C60 BHJ connects with ClAlPc/C60 PHJ directly, and this ideality factor does not mean more severe recombination in type II devices. Above numerical simulation result indicates that there are some differences in device parameters between photodiodes with one or two heterojunctions, even when these two devices have a similar FF.

Although our result shows the benefits of incorporation of ClAlPc layer between ClAlPc:C60 BHJ and C60 layer, there are still some questions on the modified p-i-p-n structure. A possible current generated in ClAlPc/C60 PHJ under illumination has a wrong direction opposite to the photocurrent generated in ClAlPc:C60 BHJ. We think this effect can be neglected because the average thickness of ClAlPc film is very small. This assumption can be confirmed by the optical absorption spectrum of two kinds of devices (shown in Fig. 6). Another question on the effect of GLAD deposited ClAlPc layer is the interfacial area between ClAlPc and C60. It has been reported that the rough interface can significantly increase the interfacial area between donor and acceptor, and therefore, improve the efficiency^10,11^. We conduct additional experiments to clarify the role of rough interface between ClAlPc (GLAD) and C60. First, we prepare some pure PHJ devices by replacing ClAlPc:C60 BHJ with ClAlPc(GLAD)/C60 PHJ. The efficiency of all these devices is less than that of the standard device A, which confirms the necessity of ClAlPc:C60 BHJ in generation of photocurrent. Second, we reduce the thickness of ClAlPc:C60 BHJ layer (≤ 25 nm) and increase the thickness of ClAlPc(GLAD)/C60 PHJ simultaneously. A typical device with the structure of ClAlPc/ClAlPc:C60 (25 nm)/ClAlPc (GLAD, 30 nm)/C60 (20 nm)/BCP/Al is denoted as device C. J-V curve and parameters of device C are drawn in Fig. 2 and listed in Table 1, respectively. Comparing to the modified device B, similar V_oc_ and FF but smaller J_sc_ result in a lower efficiency in device C. Further decreasing the thickness of ClAlPc:C60 BHJ layer and enlarging the thickness of ClAlPc(GLAD)/C60 PHJ cause even worse efficiencies. This result confirms that the ClAlPc:C60 BHJ plays a more important role on device efficiency than ClAlPc(GLAD)/C60 PHJ. Based on above experiments, we believe that the best type II device should approach the balance between a thick ClAlPc:C60 BHJ to generate charge carriers as much as possible under illumination and a thin ClAlPc(GLAD)/C60 PHJ to extract electrons as powerful as possible.

It is acknowledged that the tradeoff between the charge carrier generation power and the achievable open circuit voltage is an intrinsic limitation to further increasing the efficiency of BHJ organic solar cell^16^. Some studies have revealed a small driving force in BHJ by carefully selecting donor/acceptor organic molecules^33,34^. Our work provides a more general method to break this tradeoff by an additional PHJ. In most polymer BHJ devices, a cathode buffer layer is employed before deposition of cathode metal. Novel cathode buffer layer based on some materials, e.g., alcohol/water-soluble polymer or combination of an alcohol-soluble material layer and a BCP layer, show some benefits on polymer BHJ device performance^35,36^. Here, we suggest a new mechanism to improve the efficiency of polymer BHJ device by using a particularly deposited small molecule PHJ instead of the cathode buffer layer. Furthermore, the polymer BHJ can be prepared to absorb more light but not bear a strict transport limit, because the electron extraction from BHJ can be improved by the additional small molecule PHJ. The restriction window of the single BHJ layer in previous experiments can also be relaxed because charge extraction does not solely depends on the D/A ratio and thickness of BHJ, which in turn influences its optical absorption.

## Supplementary information

Supplementary information.
