# Supplementary material for: Improved performance of small molecule organic solar cells by incorporation of a glancing angle deposited donor layer
Source: Sci Rep. 2020 Apr 1;10:5766. doi: 10.1038/s41598-020-62769-3 (PMC7113271; doi:10.1038/s41598-020-62769-3)
Supplement: Supplementary file 1 — Supplementary information. [file 41598_2020_62769_MOESM1_ESM.docx]

Supplementary information

Improved performance of small molecule organic solar cells by incorporation of a glancing angle deposited donor layer

Qi Jiang and Yingjie Xing*

Key Laboratory for the Physics and Chemistry of Nanodevices, Beijing Key Laboratory of Quantum Devices, and Department of Electronics, Peking University, Beijing 100871, China

* xingyj@pku.edu.cn

**1 Chemical structure of ClAlPc**


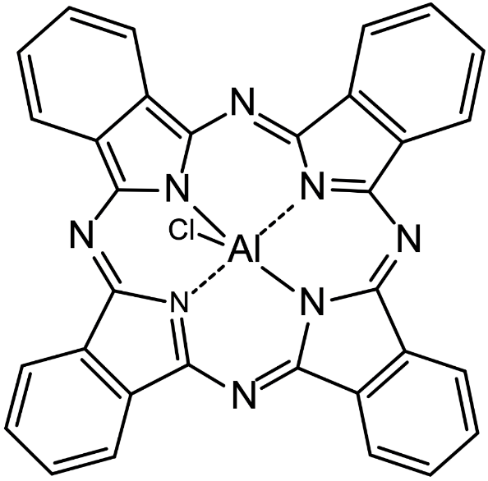


Fig. S1 Chemical structure of ClAlPc

**2 Field emission measurement**

**2.1 Calculation method**

The detail of the field emission measurement can be found in Ref. 1. According to Fowler-Nordheim theory, a better approximation for the slope of F-N plot (S), that considers the effect of the imaging force, gives

$S=-6.83\times{10}^{7}\frac{\varphi^{3/2}}{\beta}s(y)$

where *ϕ* is the work function, *β* is the local field conversion factor, *s*(*y*) is a slowly varying function with a value of 1 to 0.833; in first-order approximation, 0.917 may be used. If we assume that the value of *β* remains constant, variation of the work function (Δ*φ*) is the only reason for a changed slope (Δ*S*). Then the change of work function can be calculated by comparing the slope of F-N plot using the following Equation (*φ* and *S* are initial work function and slope, respectively).

$\varphi+\Delta\varphi=\left( \frac{S+\Delta S}{S} \right)^{\frac{2}{3}}\varphi$

In organic-organic heterojunction deposited on a sharp W tip, the change of work function of the sample reflects a small shift of the vacuum level at the sample surface after one time of deposition.

**2.2 Results**

Fermi levels of ClAlPc (5.2 eV) and C60 (4.5 eV) are used to calculate Δ*φ*. These values are adopted from Ref. 2 and 3. Values in Table S1 are used to draw Fig. 4 in the manuscript.


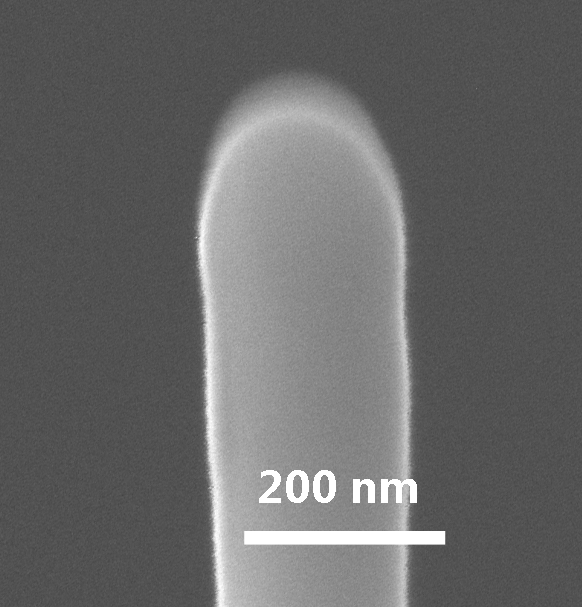


Fig. S2 SEM image of a tip after field emission measurement. The semitransparent layer covering the tip is ClAlPc/C60 bilayer, which is produced by layer-by-layer deposition. The thickness of the semitransparent layer has the magnitude of ~ 15 nm, which is determined by measured value of a quartz crystal oscillator mounted in the vacuum chamber. We find that it is difficult to take a clear SEM image within this range of thickness. This phenomenon is similar to our previous experiment. [1] We use 1 nm as a characteristic thickness for each time of deposition for simplicity.

Table S1. Band bending in ClAlPc/C60 heterojunction. Δφ is the energy difference between Fermi level and the vacuum level.

| Times of deposition (ClAlPc on C60) | Slope | Δ*φ* (eV) |
| --- | --- | --- |
| 4 | 11715 | − |
| 3 | 14351 | -0.75 |
| 2 | 16354 | -1.30 |
| 1 | 18462 | -1.84 |
| Times of deposition (C60 on ClAlPc) | Slope | Δ*φ* (eV) |
| 4 | 10149 | − |
| 3 | 10033 | -0.03 |
| 2 | 9985 | -0.04 |
| 1 | 5639 | -1.45 |

**3 Effect of GLAD deposited ClAlPc layer on dark current**

Clearly, the enlargement of V_oc_ is the prominent reason for the higher efficiency after incorporation of a GLAD deposited ClAlPc layer. In order to investigate the effect of GLAD deposited ClAlPc layer on V_oc_ without doubt, additional experiment is designed and conducted particularly. A ITO substrate is covered by ClAlPc/ClAlPc:C60 bilayer firstly. Then the substrate is rotated to the GLAD position and a mask shades part of the substrate in this position. A thin ClAlPc film is deposited in the glancing angle on only part area of the substrate, while the other part of the substrate keeps away from the evaporant. At last, the substrate is rotated back to its original horizontal position, and C60/BCP/Al layers are deposited on the whole substrate in series. We fabricate both type I and II devices on the same substrate in this way. Particularly, except the GLAD ClAlPc film in type II devices, other layers in all devices have absolutely the same thickness, microstructure, and interfacial states. Measured dark current of type I and II device on a same substrate is shown in Fig. S3. A suppressed dark current in type II device is observed because of the addition of a thin ClAlPc film.

Fig. S3 Dark current of type I and II device prepared on a same substrate. Except GLAD deposited ClAlPc film in type II device, ClAlPc/ClAlPc:C60 and C60/BCP/Al layers are all the same in two devices.

References

[1] Yingjie Xing, Shuai Li, Guiwei Wang, Tianjiao Zhao, and Gengmin Zhang, Field emission analysis of band bending in donor/acceptor heterojunction, Journal of Applied Physics 119, 245503 (2016)

[2] Toshihiko Kaji, Shiro Entani, Susumu Ikeda, and Koichiro Saiki, Origin of Carrier Types in Intrinsic Organic Semiconductors, Adv. Mater. 20, 2084–2089 (2008)

[3] S. H. Park, J. G. Jeong, H.-J. Kim, S.-H. Park, M.-H. Cho, S. W. Cho, Y.Yi, M. Y. Heo, and H. Sohn, The electronic structure of C60/ZnPc interface for organic photovoltaic device with blended layer architecture, Appl. Phys. Lett. 96, 013302 (2010)
